# Exploration of *nifH* gene through soil metagenomes of the western Indian Himalayas

**DOI:** 10.1007/s13205-015-0324-3

**Published:** 2016-01-11

**Authors:** Ravindra Soni, Deep Chandra Suyal, Santosh Sai, Reeta Goel

**Affiliations:** 1 Department of Agricultural Microbiology, College of Agriculture, Indira Gandhi Krishi Viswavidyalaya, Raipur, CG India; 2 Department of Microbiology, G.B. Pant University of Agriculture and Technology, Pantnagar, 263145 India

**Keywords:** *nif*H, Diversity, Western Himalayas, Metagenomics

## Abstract

This group has previously highlighted the prevalence of Csp genes from cold Himalayan environments. However, this study has explored the uncultured diazotrophs from metagenomes of western Indian Himalayas. The metagenomic *nif*H gene clone library was constructed from the Temperate, Subtropical and Tarai soils of Western Himalaya, India followed by polymerase chain reaction (PCR) amplification. After preliminary screening, selected clones were sequenced. In silico analysis of the clones was done, which documented 83.33 % similarities with unculturable sequence database and more than 70 % similarity with culturable bacterial database. Detailed sequence analysis of 24 *nifH* clones showed similarity to the corresponding genera of diazotrophs belonging to alpha-, beta-, gamma- and delta-proteobacteria. The prominent diazotrophs were *Azotobacter* spp., *Agrobacterium tumefaciens*, *Methylococcus capsulatus*, *Geobacter bemidjiensis*, *Dechloromonas aromatica*, *Burkholderia xenovorans, Xanthobacter autotrophicus* and *Sideroxydans* *lithotrophicus*, respectively. Alignment of these clones with culturable bacterial database suggests that most of the sequences belong to γ-proteobacterium group.

## Introduction

All N_2_ fixers carry a *nif*H gene, which encodes the Fe protein of the nitrogenase. The *nif*H database has lots of uses like phylogenetic and evolutionary analyses, the design and assessment of primers/probes, and the evaluation of nitrogenase gene diversity (Gaby and Buckley 2014); hence, making *nif*H an ideal phylogenetic gene marker for investigation of potential nitrogen-fixing organisms in natural environments (Chowdhury et al. 2009). In addition, the phylogeny based on *nif*H genes has been shown largely to resemble the 16S rRNA phylogeny (Raymond et al. 2004). This *nifH* gene has been largely studied by culture-independent approaches. Studies have provided a rapidly expanding database of *nif*H sequences and revealed a wide diversity of unculturable diazotrophs (Tan et al. 2003). These approaches provide a more complete picture of the diazotrophic community than culture-based approaches.

Further, unculturable microbial diversity could be a great resource to dig out the new ideas for sustainable agricultural practices and biotechnological applications. Therefore, in the present study, we aimed to analyze the unculturable nitrogen-fixing bacterial community of the cold adapted soil. The study area is located in north India (Uttarakhand) which comes under western Himalayan region. The *nif*H gene was amplified from metagenomic DNA of soil collected from different regions of Uttarakhand (India).

## Materials and methods

Samples were collected from the upper layer (0–15 cm) of the studied soils, from different geographic locales, namely Temperate regions of Ranichauri (78°30′E, 30°15′N, 1950 m) and Pitthoragarh (80°2′E, 29°47′N, 1967 m), Subtropical region of Chamoli (30°51′N, 79°4′, 1300 m) and Tarai region of Pantnagar (29°N, 243.8 m). Soil from at least five locations of each site was sampled, collected, composited and homogenized by sieving and stored at 4 °C till further use. Soil DNA was extracted using the Powersoil™ DNA isolation kit (Mobio Lab. Inc., Carlsbad, CA, USA) as described by the manufacturer and quantified by ultraviolet (UV) spectrophotometry at 260 nm.

The polymerase chain reaction (PCR) procedure was performed in 50 μl volumes containing 1X assay buffer with 5 mM MgCl_2_ (New England Biolabs Inc., Ipwich, MA, UK), 100 pmol of *nif*H, gene-specific universal primers (PolF—TGC GAY CCS AAR GCB GAC TC and PolR—ATS GCC ATC ATY TCR CCG GA) originally designed by Poly et al. (2001), 250 μMol of dNTPs, 1.25 U of Hot start Taq polymerase (Bangalore Genei, India), and a template DNA concentration of 50–100 ng in a pTC-150 mini-cycler PCR machine (MJ-Research, USA which is now merged in Bio-Rad) for 30 cycles (94 °C for 1 min, 55 °C for 1 min, 72 °C extension for 2 min, followed by a final extension step of 72 °C for 15 min) after initial denaturation at 95 °C for 3 min. The *nifH* amplicons were gel purified and band extracted as per the manufacturer’s instruction (AuPreP gel extraction kit, Life Technology India Pvt. Ltd., Delhi, India) and ligated into a T-cloning vector using the PCR cloning kit (Banglore Genei, India) according to the manufacturer’s instructions.

The recombinant DNA was isolated using HipurA Plasmid mini kit (Himedia, Mumbai, India) and restricted with *NcoI* enzyme. Re-amplified clones having desired size of insert were then screened out by determining their melting temperature (Tm) spectrophotometrically (Perkin Elmer 13-lambda UV–Vis spectrophotometer, Shelton, CT, USA), according to previously described method (Latha et al. 2009). Selected clones were then sent for sequencing at south campus, New Delhi. All the sequences were compared to the GENBANK database using BLAST*n* (http://blast.ncbi.nlm.nih.gov/Blast.cgi. Bethesda, MD, USA) search. Homologous sequences were retrieved from NCBI database and aligned with clone sequences using multiple sequence alignment tool clustalX (version 1.81). Further, for generic similarities the clone sequences were also aligned with 1103 available assembled eubacterial genome sequences at NCBI (http://www.ncbi.nlm.nih.gov/sutils/genom_table.cgi) database. The *nifH* sequences reported in this paper have been deposited in the GenBank database under accession numbers GU117589 to GU117602 and GU121497 to GU121506, respectively.

## Results and discussion

A 360-bp amplicon was obtained from PCR amplification. After successive cloning of *nif*H gene amplicons, a total of 60 clones were obtained from four soil samples. The clones were further screened by determining Tm (data were not shown). One representative from each group of clones having similar or nearer Tm value was taken for sequencing. In total 24 *nifH* clones were selected and sequenced. Out of 24 clones 14 from Pithoragarh soil (clones name annoted by PN), 4 from Ranichauri soil (RN) and 3 each from Pantnagar (PPN) and Chamoli soil (CN). Results revealed that majority of nitrogen fixer are from unculturable community. Here, total 83.33 % (20/24) clones documented more than 85 % similarities with unculturable bacterial sequences available at NCBI database. Further, Clones PN 4 and PN 11 show only 87 and 89 % similarities with unculturable bacterium clone, and remaining of the clones have more than 90 % similarity with unculturable clone.

However, these clones were also aligned with 1103 assembled bacterial genome database (http://www.ncbi.nlm.nih.gov/sutils/genom_table.cgi) where more than 70 % similarities were considered for generic confirmation. The results suggest that the clones which are showing similarities with the unculturable bacterium clones were also depicting similarity with assembled bacterial (culturable) genome database, but the percentage of similarity is less than unculturable bacterium clone (Table 1). All clone sequences belong to proteobacteria group, where dominance is from Gama subdivision (11/24) followed by beta (6/24), alpha (4/24) and delta (3/24) subsequently. Similarly, some recent work on western Himalayan soils also reported the dominance of Proteobacteria group (Gangwar et al. 2009; Yadav et al. 2015). Further, genome similarities’ search results revealed that 24 sequences represent 13 different sequence types among which 5 clones show 79–95 % identity to *Azotobacter* spp. Regardless that cyanobacterium is also a major part of soil nitrogen fixer; none of the sequences depicts similarities with culturable cyanobacteria. However, two clones, i.e. PN8 and PPN6, have the similarities with unculturable *cyanobacterium* clone.

**Table 1:** Sequence similarity results of *nif*H clones with culturable and unculturable bacterial sequence database

Clone	Similarities with unculturable bacterial database			Similarities with culturable bacterial database		
	Unculturable bacterial clone name	*E* value	Similarities	Culturable bacterial genera	Similarities	*E* value
PN4	Uncultured bacterium clone Qinglin-5	5e−153	87	*Azotobacter vinelandii *DJ	79	1e−103
PN5	Uncultured bacterium clone IPA108	5e−145	92	*Xanthobacter autotrophicus*	88	4e−96
PN8	Uncultured soil bacterium clone T4t035	7e−143	94	*Azoarcus* sp. BH72	89	2e−110
PN9	Uncultured bacterium clone LM108	1e−165	97	*Azotobacter vinelandii*	94	3e−155
PN11	Uncultured bacterium clone pCPS202	3e−129	89	*Geobacter bemidjiensis*	88	2e−122
PN12	–	–	–	*Agrobacterium tumefaciens*	97	6e−171
PN15	Uncultured bacterium clone LM108	4e−171	97	*Pseudomonas stutzeri* A1501	87	3e−115
PN19	Uncultured nitrogen-fixing	5e−170	98	*Azotobacter vinelandii* DJ	95	4e−149
PN24	Uncultured bacterium clone LM108	2e−170	98	*Azotobacter chroococcum*	98	3e−173
PN25	Uncultured bacterium clone S2j	6e−163	95	*Rhodospirillum centenum* SW	91	2e−136
PN27	Uncultured soil bacterium clone TC07	7e−150	92	*Sideroxydans lithotrophicus*	84	7e−100
PN29	–	–		*Dechloromonas aromatica*	93	1e−152
PN30	Uncultured bacterium clone LM108	3e−168	96	*Agrobacterium tumefaciens*	96	3e−169
PN31	Uncultured bacterium clone LM108	4e−171	98	*Azotobacter vinelandii*	95	1e−163
RN3	–	–		*Agrobacterium tumefaciens*	99	4e−177
RN7	–	–		*Dechloromonas aromatica*	95	3e−154
RN15	Uncultured cyanobacterium clone FAL	2e−130	92	*Burkholderia xenovorans*	88	2e−109
RN19	Uncultured bacterium clone Yushu-15	6e−119	87	*Geobacter bemidjiensis*	87	1e−114
PPN2	Uncultured soil bacterium clone T1t065	1e−133	91	*Xanthobacter autotrophicus*	87	5e−108
PPN3	Uncultured bacterium clone Yushu-15	6e−131	90	*Geobacter* sp. M18	89	2e−115
PPN6	Uncultured soil bacterium clone T1t065	1e−146	94	*Burkholderia xenovorans*	88	7e−114
CN5	Uncultured soil bacterium clone T4t035	2e−150	94	*Azoarcus* sp. BH72	85	3e−99
CN6	Uncultured soil bacterium clone T1t065	7e−131	90	*Methylococcus capsulatus*	85	7e−100
CN8	Uncultured soil bacterium clone T1t065	1e−133	91	*Methylococcus capsulatus*	86	1e−101

Further, *Agrobacterium tumefaciens* is the only representative of symbiotic nitrogen fixers present among the clone sequences. Moreover, clone PN8 (89 %) and CN5 (85 %) have the generic similarity with *Azoarcus*, which is a rice obligate endophytes and fix the atmospheric nitrogen (Wartiainen et al. 2008). However, these two clones also depict more than 95 % similarity with unculturable bacterial clone. The point of interest is that clones PN29 (Pithoragarh) and RN7 (Ranichauri) have the similarities with genus *Dechloromonas* which is a unique genus with a broad range of novel metabolic capabilities and bioremediative applicability. One more clone, PN27, shows the 84 % similarities with *nif*H gene of *Sideroxydans lithotrophicus*, recently described Fe(II)-oxidizing bacteria, and belongs to Xanthomonadaceae family. In the *nifH* clone library clones CN6 and CN8 belong to *Methylococus capsulatus* (methanogen, gamma-proteobacteria) and *Methylococus* is an unusual genus because it shares properties of both type I and II, Methanogens (Whittenburry and Dalton 1981). The methanotrophic bacteria had been identified from rice root, freshwater lake, termite gut, Douglas fir soil site and an oligotrophic ocean (Ueda et al. 1995). Subsequently, several studies have indicated that structural and functional diversity of rhizosphere population is affected by the plant species due to difference in root exudation and rhizo-deposition in different root zones (Kent and Triplett 2002). Further, soil type, growth stage of plant, cropping practices and environmental factors influence the composition of the microbial community in the rhizosphere (Wieland et al. 2001). Nonetheless, two clones namely RN15 and PPN6 were having the 88 % similarities with *Burkholdaria xenovorans*. This bacterium is a plant-associated nitrogen fixer. Many nitrogen-fixing *Burkholderia s*pp. are reported earlier from different plants (rice, maize, sugar cane, sorghum, coffee and tomato) or from their rhizospheres. Some of them are *B. unamae*, *B. xenovorans*, *B. silvatlantica*, *B. tropica*, *B. tuberum*, *B. phymatum*, *B. mimosarum* and *B. Nodosa* (Caballero-Mellado et al. 2007). Further, clones PN5 (88 %) and PPN2 (87 %) have the similarities with *Xenthobacter autotrophicus*, a hydrogen-using bacterium. Remaining clones belongs to common soil bacteria i.e. *Geobacter* (PN11, RN19 and PPN3), *Pseudomonas* (PN15) and *Rhodospirillum* (PN25), respectively. However, PPN3 also has the similarity with some *Bacillus* species.

In this study, we found that the active diazotrophic community varied strongly between various soil types collected from different geographic locations. However, the similar sequence types were also found at different soil types, and the majority of the sequences clustered with *Azotobacter* species and gamma-proteobacteria group. Presence of bacterial community belonging to proteobacteria group assures the capability of N_2_ fixing in rhizospheres of these soils. Similarly, Yadav et al. (2015) reported variation in bacterial community after phylogenetic analysis of western Himalaya soils, which revealed that 82 distinct species of 31 different genera belonged to four phyla Actinobacteria, Bacteroidetes, Firmicutes and Proteobacteria. Since, the process of N_2_ fixing is not only dependent on bacterial type but also on characteristics of soils; therefore, further study can explore the effect of presence of these diversifying communities on soil fertility and crop productivity.
